# A Dimerization-Dependent Mechanism Drives the Endoribonuclease Function of Porcine Reproductive and Respiratory Syndrome Virus nsp11

**DOI:** 10.1128/JVI.03065-15

**Published:** 2016-04-14

**Authors:** Yuejun Shi, Youwen Li, Yingying Lei, Gang Ye, Zhou Shen, Limeng Sun, Rui Luo, Dang Wang, Zhen F. Fu, Shaobo Xiao, Guiqing Peng

**Affiliations:** a State Key Laboratory of Agricultural Microbiology, Huazhong Agricultural University, Wuhan, China; b College of Veterinary Medicine, Huazhong Agricultural University, Wuhan, China; c Pathology Department, College of Veterinary Medicine, University of Georgia, Athens, Georgia, USA; d The Cooperative Innovation Center for Sustainable Pig Production, Huazhong Agricultural University, Wuhan, Hubei, China

## Abstract

Porcine reproductive and respiratory syndrome virus (PRRSV) RNA endoribonuclease nsp11 belongs to the XendoU superfamily and plays a crucial role in arterivirus replication. Here, we report the first crystal structure of the arterivirus nsp11 protein from PRRSV, which exhibits a unique structure and assembles into an asymmetric dimer whose structure is completely different from the hexameric structure of coronavirus nsp15. However, the structures of the PRRSV nsp11 and coronavirus nsp15 catalytic domains were perfectly superimposed, especially in the “active site loop” (His129 to His144) and “supporting loop” (Val162 to Thr179) regions. Importantly, our biochemical data demonstrated that PRRSV nsp11 exists mainly as a dimer in solution. Mutations of the major dimerization site determinants (Ser74 and Phe76) in the dimerization interface destabilized the dimer in solution and severely diminished endoribonuclease activity, indicating that the dimer is the biologically functional unit. In the dimeric structure, the active site loop and supporting loop are packed against one another and stabilized by monomer-monomer interactions. These findings may help elucidate the mechanism underlying arterivirus replication and may represent great potential for the development of antiviral drugs.

**IMPORTANCE** Porcine reproductive and respiratory syndrome virus (PRRSV) is a member of the family Arteriviridae, order Nidovirales. PRRSV is a major agent of respiratory diseases in pigs, causing tremendous economic losses to the swine industry worldwide. The PRRSV nsp11 endoribonuclease plays a vital role in arterivirus replication, but its precise roles and mechanisms of action are poorly understood. Here, we report the first dimeric structure of the arterivirus nsp11 from PRRSV at 2.75-Å resolution. Structural and biochemical experiments demonstrated that nsp11 exists mainly as a dimer in solution and that nsp11 may be fully active as a dimer. Mutagenesis and structural analysis revealed NendoU active site residues, which are conserved throughout the order Nidovirales (families Arteriviridae and Coronaviridae) and the major determinants of dimerization (Ser74 and Phe76) in Arteriviridae. Importantly, these findings may provide a new structural basis for antiviral drug development.

## INTRODUCTION

Nidoviruses are enveloped, positive-sense, single-stranded RNA [(+)ssRNA] viruses comprising the families Arteriviridae, Coronaviridae, Mesoniviridae, and Roniviridae (1–4). Nidoviruses (Arteriviridae and Coronaviridae) are important pathogens that cause significant diseases in animals and humans, typically causing respiratory and enteric disease (5, 6). The genome length of Arteriviridae members is approximately 12.7 to 15.7 kb, among the “small-genome nidoviruses” (7). Coronaviridae and Roniviridae belong to a group of “large-genome nidoviruses,” as their genome lengths span 26.3 kb to 31.7 kb (1), whereas members of the Mesoniviridae have medium-sized (16-to-200-kb) genomes, between those of small- and large-genome nidoviruses (3, 4). Nevertheless, all nidoviruses are grouped together due to their similar replication/transcription strategies and their relatively close genetic relationship (1, 8).

Porcine reproductive and respiratory syndrome virus (PRRSV) is a member of the family Arteriviridae, which also includes equine arteritis virus (EAV), lactate dehydrogenase-elevating virus (LDV) of mice, and simian hemorrhagic fever virus (SHFV) (7). PRRSV is the causative agent of porcine reproductive and respiratory syndrome (PRRS), which has become one of the most important infectious diseases in the swine industry and causes tremendous economic losses worldwide (9, 10). The PRRSV genome is approximately 15 kb in length, with 10 overlapping open reading frames (ORFs), and consists of both nonstructural genes (ORF1a and ORF1b) and structural genes (ORF2 to ORF7) (7, 11, 12). ORF1a and ORF1b comprise approximately 80% of the viral genome and encode at least 16 nonstructural proteins (nsps), including nsp1α, nsp1β, nsp2, nsp2TF/nsp2N, nsp3 to nsp6, nsp7α, nsp7β, and nsp8 to nsp12 (13); ORF2 to ORF7 encode the viral structural proteins GP2, E, GP3, GP4, GP5, ORF5a, M, and N (14, 15). The resulting mature nsps direct viral genome replication and subgenomic mRNA transcription via a membrane-anchored replicase/transcriptase complex, and these mRNAs are then translated to produce structural and accessory proteins (12). nsp9 (RNA-dependent RNA polymerase) and nsp10 (helicase) (7) are the key replicative enzymes in the replicase/transcriptase complex; nsp11 (endoribonuclease) may also play a key role in arterivirus replication, though the exact function of endoribonucleases in nidovirus replication remains unclear (7, 16).

PRRSV nsp11 possesses nidovirus uridylate-specific endoribonuclease (NendoU) activity, which is important for arterivirus replication (2). XendoU, reported to be an endoribonuclease in eukaryotes, is involved in processing the intron-encoded box C/D U16 small nuclear RNA from its pre-mRNA (17). XendoU and NendoU, which specifically cleaves 5′ uridine nucleotides of RNA substrates to generate a 2′-3′-cyclic phosphate end product, possess common functional characteristics (18–20). The endoribonuclease activity of coronavirus (CoV) nsp15 and arterivirus nsp11 has been confirmed (19–21), and recombinant arterivirus nsp11 displays broad substrate specificity *in vitro* (20). Moreover, the NendoU activity of coronavirus nsp15 is stimulated by Mn^2+^ (19, 21, 22), whereas Mn^2+^ was reported to be inhibitory to the activity of arterivirus nsp11 NendoU (20). The crystal structures of severe acute respiratory syndrome coronavirus (SARS-CoV) nsp15 and murine hepatitis virus (MHV) nsp15 show that the biological unit of nsp15 is a hexamer (19, 21) and that the N-terminal domain (NTD) is important for oligomerization (23). Although NendoU activity is common to nidoviruses (Arteriviridae, Coronaviridae, and Roniviridae), the NendoU domains exhibit considerable variation (20). There is no detailed structural information to date for arterivirus nsp11.

In this study, we performed structural and functional analyses of nsp11 to elucidate the mechanism underlying the function of PRRSV endoribonuclease nsp11 during arterivirus replication and to identify potential drug targets for controlling PRRS disease. We report the crystal structure of PRRSV endoribonuclease nsp11 and demonstrate that the folding of NendoU active site residues is widely conserved among members of the order Nidovirales (families Arteriviridae and Coronaviridae). Our data also indicate that nsp11 is fully active as a dimer, and we elaborate on the structural basis underlying this finding.

## MATERIALS AND METHODS

### Plasmid construction.

The sequence encoding the 223-residue nsp11 gene corresponds to nucleotides 10851 to 11520 in the genome of the PRRSV WUH3 strain (GenBank accession no. HM853673) (24). For crystallization, wild-type and mutant (K173A) nsp11 gene sequences were cloned by PCR amplification into pET-42b (+) with a C-terminal His_6_ tag using the NdeI and BamH I restriction sites. Mutant plasmid (K173A) was used as the template for the generation of expression constructs encoding mutant (S74A, F76A, and R153A) nsp11 derivatives for dimerization experiments. Meanwhile, to obtain high expression in prokaryotic cells, wild-type nsp11, flanked by an N-terminal His_6_ tag and S tag and a C-terminal His_6_ tag, was cloned into pET-30a (+) between the EcoR I and XhoI restriction sites. In addition, to obtain high expression in eukaryotic cells, wild-type nsp11, flanked by an N-terminal hemagglutinin (HA) tag, was cloned into pCAGGS vector using the EcoRI and XhoI restriction sites. Point mutations (S74A, F76A, H129A, K173A, T177A, and Y219A) were engineered using overlap extension PCR, and the fragments were cloned into pET-30a (+) and pCAGGS vector according to the same method. All constructs were validated by DNA sequencing.

### Protein expression and purification.

For analysis of wild-type nsp11 expression, the recombinant plasmids were transformed into Escherichia coli strain Trans BL21(DE3) pLysS (Beijing TransGen Biotech Co., Ltd.). Transformed cells were cultured at 37°C in LB medium containing 50 μg/ml kanamycin. Induction with 0.8 mM IPTG (isopropyl β-d-1-thiogalactopyranoside) was performed when the culture density reached an optical density at 600 nm (OD_600_) of 0.6 to 0.8, and cell growth continued for an additional 1 h at 37°C. For analysis of the expression of the nsp11 mutant proteins, the recombinant plasmids were transformed according to the same method. When the cells reached an OD_600_ of 0.6 to 0.8, IPTG was added to give a final concentration of 0.8 mM. Then, the cells were grown for an additional 5 h at 37°C before harvesting. To solve the phase problem, a selenomethionine (Se-Met)-labeled nsp11 mutant (K173A) was expressed in Trans BL21(DE3) pLysS using M9 salt medium (Qingdao Rishui Biological Technology Corporation) supplemented with 50 μg/ml kanamycin, 0.4% glucose, 2 mM MgSO_4_, and 0.1 mM CaCl_2_ at 37°C until an OD_600_ of 0.8 was reached. Then, the amino acid mixture (100 mg lysine, phenylalanine, and threonine per liter; 50 mg isoleucine, leucine, and valine per liter; and 60 mg selenomethionine per liter) was added 15 min before induction. IPTG was added to give a final concentration of 0.8 mM, and the cells were grown for an additional 5 h at 37°C before harvesting.

For protein purification, cells were harvested by centrifugation at 8,500 rpm for 5 min in a high-speed refrigerated centrifuge (CR-21G; Hitachi), resuspended with phosphate-buffered saline (PBS; 137 mM NaCl, 3 mM KCl, 10 mM Na_2_HPO_4_·12H_2_O, and 2 mM KH_2_PO_4_, pH 7.4) and lysed by passage through an AH-1500 homogenizer (ATS Engineering Inc.) at 15,000 lb/in^2^. After centrifugation at 8,500 rpm for 40 min, the supernatant was filtered with a 0.45-μm-pore-size filter and loaded onto a nickel-charged HisTrap HP column (GE Healthcare). The proteins were eluted with elution buffer (20 mM Tris-HCl, 1 M NaCl, and 500 mM imidazole, pH 7.4). The harvested protein was then concentrated to approximately 2.0 ml and filtered using a Superdex200 gel filtration column (GE Healthcare) equilibrated with buffer (20 mM Tris-HCl and 1 M NaCl, pH 7.4). For crystallization, the purified protein was concentrated to approximately 8 mg/ml, flash-frozen with liquid nitrogen, and stored at −80°C. The concentration of the purified PRRSV nsp11 was determined by the absorbance at 280 nm (*A*_280_) using a NanoDrop 2000c UV-Vis spectrophotometer (Thermo Fisher Scientific).

### Crystallization, data collection, and structure determination.

Crystallization screens for wild-type nsp11 and the K173A mutant protein at a concentration of 8 mg/ml were performed via the hanging-drop vapor-diffusion method at 20°C. Crystals of the Se-Met derivative (K173A) mutant were obtained to solve the phase problem. The crystallization conditions were optimized, and the best crystals for both wild-type and Se-Met-labeled nsp11 were obtained by vapor diffusion in hanging drops consisting of 3 μl of reservoir solution (0.2 M sodium citrate tribasic dehydrate [pH 5.3] and 8% [wt/vol polyethylene glycol 3350) and 3 μl of concentrated protein solution (8 mg/ml)–20 mM Tris-HCl–1 M NaCl (pH 7.4), followed by incubation at 20°C for 5 days (wild-type nsp11) or 7 days (Se-Met-labeled nsp11). Then, the crystals were flash-cooled in liquid nitrogen in a cryoprotectant solution containing 30% ethylene glycol and 70% reservoir solution (0.2 M sodium citrate tribasic dehydrate [pH 5.3] and 9.6% [wt/vol] polyethylene glycol 3350). Data collection was performed at the Shanghai Synchrotron Radiation Facility (SSRF) with a BL17U1 beam line (wavelength = 0.97910 Å, temperature = 100K). Reflections were integrated, merged, and scaled using HKL-3000 (25), and the resulting statistics are listed in Table 1. The structure of nsp11 was solved by the use of the single-wavelength anomalous dispersion (SAD) method and a Se-Met derivative K173A mutant. All three potential selenium atoms in the nsp11 monomer were located, and the initial phases were calculated using the AutoSol program from the PHENIX software suite (26). Manual model rebuilding was performed using COOT (27) and then refined in the PHENIX software suite.

**TABLE 1:** Statistics of data collection and refinement

Parameter	Value(s)^*a*^	
	Wild-type nsp11	Mutant Se-Met nsp11 K173A
Data collection statistics		
Space group	P4_1_2_1_2	P4_1_2_1_2
Cell parameter [a, b, c (Å)]	77.317, 77.317, 204.630	77.353, 77.353, 204.19
ϵμσ>α, β, γ (°)	90.0, 90.0, 90.0	90.0, 90.0, 90.0
Wavelength (Å)	0.97910	0.97910
Resolution (Å) (range)	50.00–2.75	50.00–2.65
No. of reflections	17,000	18,849
Completeness (%)	99.9 (100.0)	99.8 (100.0)
*R*_merge_^*b*^ (%)	0.110 (0.80)	0.070 (0.504)
*I*/δ (last shell)	24.43 (5.69)	29.73 (7.79)
Redundancy (last shell)	7.3 (7.7)	7.6 (7.7)
Refinement statistics		
Resolution (Å) (range)	42.66–2.75	
No. of reflections	16,931	
*R*_work_/*R*_free_^*c*^ (%)	22.8/28.6	
No of protein atoms	3383	
No. of solvent atoms	17	
RMSD		
Bond length (Å)	0.010	
Bond angle (°)	1.323	
Avg B factor (Å^2^)	56	
Ramachandran plot: core, allow, disallow (%)	94.3, 5, 0.7	

a The highest-resolution values are indicated in parentheses.

b *R*_merge_ = ∑∑   *I*_i_ − ﹤*I*﹥  /∑∑*I*_i_ (where *I*_i_ is the intensity measurement of reflection h and ﹤I﹥ is the average intensity from multiple observations).

c *R*_work_ = ∑||*F*_o_| − |*F*_c_||/∑|*F*_o_| (where *F*_o_ and *F*_c_ are the observed and calculated structure factors, respectively; *R*_free_ is equivalent to *R*_work_, but 5% of the measured reflections have been excluded from the refinement and set aside for cross-validation.

### Structural analysis, sequence alignment, and phylogenetic reconstruction.

Detailed molecular interactions between the two monomers of nsp11 were determined using LIGPLOT (28), and the other structure figures were generated using PyMOL (Schrödinger). The buried surface areas between the two monomers and the root mean square deviation (RMSD) were analyzed using PDBePISA (http://pdbe.org/pisa/) and PDBeFold (http://pdbe.org/fold/), respectively. Additionally, cell content analysis was generated using the CCP4 suite (29). The amino acid sequences of arterivirus nsp11 and coronavirus nsp15 were aligned using ClustalW2 (30) and visualized with the ESPript 3 server (http://espript.ibcp.fr) (31). The phylogenetic relationships were analyzed using the maximum likelihood algorithm in the MEGA package (32). The analyzed viruses (abbreviations; NCBI accession numbers) were as follows: HCoV-HKU1 (human coronavirus HKU1; YP_173236), SARS-CoV (SARS coronavirus wtic-MB; AGT21317), MHV (murine hepatitis virus strain A59; NP_740619), PEDV (porcine epidemic diarrhea virus; AIM47753), HCoV-229E (human coronavirus 229E; AGT21366), TGEV (transmissible gastroenteritis virus virulent Purdue; ABG89333), FIPV (feline infectious peritonitis virus; AGZ84515), PRRSV (porcine respiratory and reproductive syndrome virus strain WUH3; ADO33722), EAV (equine arteritis virus; NP_705592), SHFV (simian hemorrhagic fever virus; AHH54245), and LDV (lactate dehydrogenase-elevating virus; AAA74104).

### *In vitro* dimerization experiments.

As the oligomeric state of the mutant [K173A; pET-42b (+)] nsp11 protein is the same as that of the wild type (data not shown), the mutant (K173A) protein was purified for size exclusion experiments because the expression of wild-type nsp11 was low. Oligomerization of wild-type (1 mg) and mutant (S74A, F76A, and R153A) (1 mg) nsp11 proteins was analyzed using a Superdex 75 10/300 GL column (GE Healthcare) with a buffer containing 20 mM Tris-HCl (pH 7.4) and 200 mM NaCl at a flow rate of 0.6 ml/min (4°C). Wild-type and mutant (S74A, F76A, and R153A) nsp11 proteins eluted in different fractions were analyzed by SDS-PAGE. Equal volumes (200 μl) of Bio-Rad size exclusion standards (catalog no. 151-1901; 1 ml/vial), conalbumin (75 kDa; 2.5 mg) (GE Healthcare), and mutant (G1A, L54A, R55A, Y69A, G137A, G138A, V165A, and S166A) nsp11 proteins (1 mg) were analyzed under the same buffer conditions. Additionally, sedimentation velocity analysis was carried out using an XL-A model centrifuge (Proteome Lab) at 45,000 rpm and 18°C in 400-μl double-sector cells. The sedimentation boundary was monitored every 3 min using a wavelength of 280 nm for a total of 110 scans. Data were interpreted with the model-based distribution of Lamm equation solutions [c(s)] using Sedfit software (33). The obtained results were analyzed using Origin 8.0 software. The predicted weight-averaged molar masses were calculated using DNASTAR (version7.1) software.

### Enzyme activity assay.

The RNA substrate (5′-6-carboxyfluorescein [FAM]-dA-rU-dA-dA-6-carboxytetramethylrhodamine [TAMRA]-3′) was chemically synthesized by the GenScript Corporation. The endoribonuclease activity of nsp11 was examined using fluorescence resonance energy transfer (FRET) following a previously published protocol (34). The assay was performed with 2 μM enzyme and 1 μM fluorescent substrate at 25°C in buffer containing 50 mM HEPES (pH 7.5), 50 mM KCl, and 1 mM dithiothreitol (DTT) diluted with 0.1% diethyl pyrocarbonate-treated water. The activity was assayed with an excitation wavelength of 492 nm and an emission wavelength of 518 nm using a fluorescence time scan on a Fluoroskan Ascent instrument (Thermo Labsystems, Helsinki, Finland) and was recorded every 15 min for 60 min. The activity assays for the wild-type and mutant (S74A, S76A, H129A, K173A, T177A, and Y219A) nsp11 proteins were performed under the same conditions. It should be mentioned that purification of the mutant (H144A, R153A, D180A, and D204A) proteins was unsuccessful because these mutations rendered the protein insoluble. Experiments were performed in triplicate, and the values (± standard deviations [SD]) of the results of triplicate experiments are shown. In addition, the wild-type and mutant (S74A, S76A, H129A, K173A, T177A, and Y219A) nsp11 proteins were analyzed by SDS-PAGE.

### Luc reporter gene assays.

HEK293T cells were seeded into 48-well plates and incubated until the cells reached approximately 80% confluence. Then, the cells were cotransfected with 0.1 μg of the reporter plasmid (beta interferon-luciferase [IFN-β–Luc] or IRF3-Luc), 0.02 μg of plasmid pRL-TK (Promega) encoding Renilla luciferase, 0.4 μg of wild-type plasmid, or 0.4 μg of mutant (S74A, F76A, H129A, K173A, T177A, and Y219A) plasmids using Lipofectamine 2000. Total transfected DNAs were equalized to 0.52 μg by the addition of empty pCAGGS vector. At 24 h after the initial transfection, the cells were infected with Sendai virus (SEV). At 40 h posttransfection, the cells were harvested and the luciferase activity was measured using a Dual-Luciferase reporter assay system (Promega) on a GloMax 20/20 luminometer reader (Promega). Firefly luciferase activity was normalized to Renilla luciferase. Experiments were performed in triplicate, and statistical significance was determined using an unpaired two-tailed Student's *t* test. Values of <0.05 were considered statistically significant.

### Western blot analysis.

Briefly, to analyze the expression levels of the wild-type and mutant (S74A, F76A, H129A, K173A, T177A, and Y219A) nsp11 proteins, HEK293T cells were transfected with various plasmids using the same method. At 40 h posttransfection, cells were harvested by adding lysis buffer (Beyotime), and the protein concentration was measured and adjusted. The same amounts of each protein sample were then analyzed by Western blotting with anti-HA antibody (Ab; Sigma). Expression of GAPDH (glyceraldehyde-3-phosphate dehydrogenase) was detected with anti-GAPDH monoclonal Ab (MAb) (Sigma) to confirm loading of equal protein amounts.

### Cell viability assay.

HEK293T cells cultured in white 96-well plates (Corning, Tewksbury, MA, USA) were transfected with an empty vector or wild-type or mutant (S74A, F76A, H129A, K173A, T177A, and Y219A) nsp11 plasmids (0.2 μg) using Lipofectamine 2000. In addition, the different doses of the wild-type plasmid (0 to 0.3 μg) were transfected, and total transfected DNAs were equalized to 0.3 μg by the addition of empty pCAGGS vector. At 40 h posttransfection, cell viability was evaluated using CellTiter-Glo luminescent cell viability assay reagent (Promega, Madison, WI, USA) following the manufacturer's protocol. Briefly, an equal volume (100 μl) of CellTiter-Glo reagent was added and the reaction mixture was shaken for 2 min on an orbital shaker and incubated for a further 10 min at room temperature. The luminescence of each well was measured on a 1450 MicroBeta TriLux instrument (PerkinElmer, Waltham, MA, USA). The percentage of cell viability was calculated as follows: percentage of cell viability = 100 × (luminescence of the experimental group/luminescence of the control group). Experiments were performed in triplicate, and statistical significance was determined using an unpaired two-tailed Student's *t* test. Values of <0.05 were considered statistically significant.

### Protein structure accession number and statistical analysis.

Coordinates and structure factors for PRRSV nsp11 were deposited in the RCSB Protein Data Bank under accession number 5DA1.

## RESULTS AND DISCUSSION

### Crystal structure of PRRSV endoribonuclease nsp11.

Previous studies demonstrated that full-length wild-type endoribonucleases (SARS-CoV nsp15, MHV nsp15, EAV nsp11, and PRRSV nsp11) are expressed only weakly; accordingly, these endoribonucleases may be toxic to E. coli and cause slow cell growth and low protein yields (20, 21, 23). Thus, to obtain wild-type nsp11, we assessed different expression vectors and used different E. coli strains as hosts; nevertheless, the yield of wild-type nsp11 remained extremely low. Indeed, our experimental results showed that wild-type nsp11 causes cell cytotoxicity and death after an extended expression time (approximately 3 h). The duration of wild-type nsp11 expression was examined at different times (0 min, 15 min, 30 min, 45 min, 60 min, and 90 min) at 37°C, with 60 min found to be the best expression time (data not shown). The yields of wild-type nsp11 from the expression vectors [pET-42b (+) and pET-30a (+)] were estimated to be approximately 0.05 mg and 0.2 mg of protein, respectively, per liter of bacterial cell culture. In contrast, the yield of mutant protein reached approximately 5 to 6 mg/liter (37°C, 5 h). Previous studies indicated that functional endoribonucleases can cleave the 3′ terminus of the pyrimidines of ssRNA and dsRNA substrates (18, 20), which might act on both their own and cellular mRNA and cause NendoU expression to be potentially “suicidal” (7).

**FIG 1:**
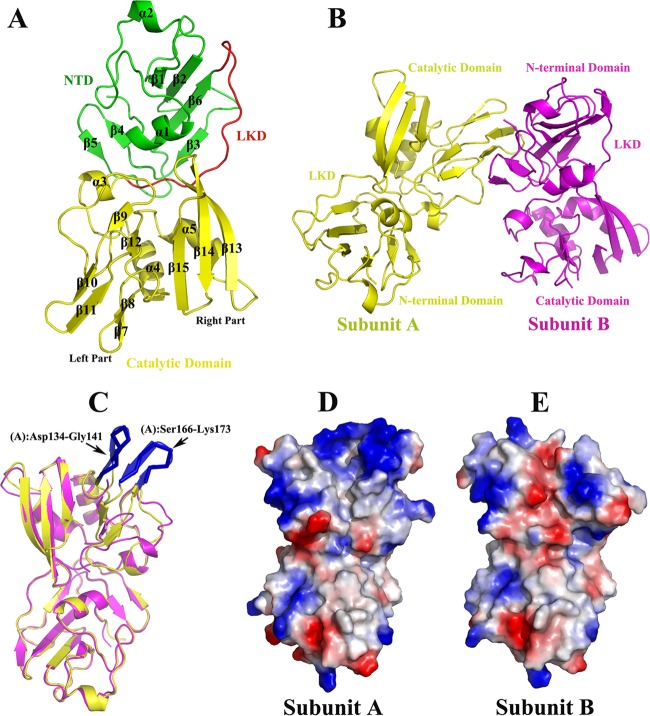
The structure of PRRSV nsp11. (A) Overall structure of the nsp11 monomer. The monomer structure of nsp11 (subunit A) is shown as a colored cartoon. The N-terminal domain (NTD), linker domain (LKD), and catalytic domain are colored green, red, and yellow, respectively. (B) Contents of the asymmetric unit. The two monomers (designated A and B) are colored yellow and magenta, respectively. The NTD, LKD, and catalytic domain are marked as described for panel A. (C) Superimposition of the two monomers of the asymmetric unit. The two monomers are colored using the same scheme as described for panel B. The missing residues (Asp134 to Gly141 and Ser166 to Lys173) are highlighted with blue ribbons in subunit B. (D and E) Subunits A and B are shown as a molecular surface model colored according to electrostatic potential (red for negatively charged regions and blue for positively charged regions). The side views of the molecular surface of nsp11 are shown at the same angles (C, D, and E).

The crystal structure of PRRSV nsp11 (residues Gly1 to Glu223) was determined using the SAD method and was refined to 2.75-Å resolution, which was of sufficient quality to trace the entire chain (excluding the C-terminal His_6_ tags). The Matthews coefficient and solvent content are 2.99 and 58%, respectively, as determined by cell content analysis. The solvent content value is high, which may explain why the diffraction of the crystals was poor. The crystal belongs to the space group P4_1_2_1_2 and consists of two subunits in an asymmetric unit (Fig. 1B). Interestingly, subunit A is visibly different from subunit B (Fig. 1C, D, and E). In subunit B, residues in the regions Asp134-Gly141, Ser166-Lys173, and Leu222-Glu223 could not be traced due to a lack of interpretable electron density (Fig. 1C). The crystal structure can be divided into two major parts: the N-terminal domain (NTD; Gly1 to Phe90) and the C-terminal catalytic domain (Arg107 to Glu223). The NTD is formed by six β-strands (β1 to β6) and two α-helices (α1 to α2) and is connected to the catalytic domain through a linker domain (LKD; Val91 to Thr106). The catalytic domain has a typical fold consisting of a compact groove region containing sequentially connected left and right parts (Fig. 1A): the left part of the catalytic domain consists of two α-helices (α3 and α4) and six β-strands (β7 to β12), and the right part is formed by three antiparallel β-strands (β13 to β15) and one α-helix (α5). Details of the data collection and structure refinement are summarized in Table 1.

### Mutational studies in the dimerization interface.

**FIG 2:**
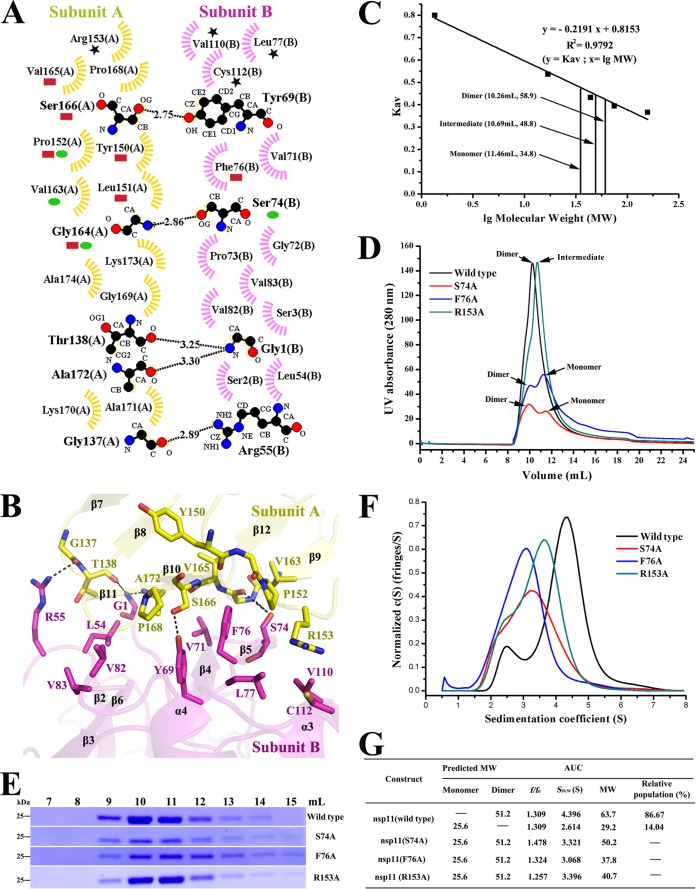
Dimerization mutants disrupt nsp11 dimerization in solution. (A) All the interactional residues between subunits A (yellow) and B (magenta) were determined using LIGPLOT. The residues that interact with Ser74, Phe76, and Arg153 are denoted with ovals (green), rectangles (red), and pentacles (black), respectively. Carbon, nitrogen, and oxygen atoms are shown as black, blue, and red circles, respectively. Hydrogen bond interactions are shown as black dashed lines between the respective donor and acceptor atoms, along with the bond distance. Hydrophobic interactions are indicated by arcs with spokes radiating toward the atoms they contact. (B) Dimerization interface of nsp11. The partial interactional residues (including interactions with Ser74, Phe76, and Arg153) between subunits A and B are shown with a stick. The figure is colored as described for panel A. (C) Calculated molecular weights of the nsp11 protein peaks with the values obtained for known calibration standards (Bio-Rad and GE Healthcare). The calculated molecular weight of nsp11 peaks was determined by fitting to the calibration curve (Kav = volumes of elution [Ves]/24); volumes of elution of 10.26 ml (approximately 58.9), 10.69 ml (approximately 48.8), and 11.46 ml (approximately 34.8) are indicated by arrows. (D) Size exclusion experiment with the nsp11 wild type and mutants (S74A, F76A, and R153A). The calculated molecular masses were determined by fitting to the calibration curve as described for panel C. The wild type is colored black, and the mutants (S74A, F76A, and R153A) are colored red, blue, and bottle green, respectively. (E) SDS-PAGE analysis of wild-type and mutant (S74A, F76A, and R153A) nsp11. The elution volume is labeled as described for panel D. Molecular mass markers are shown. (F and G) Sedimentation velocity analysis of wild-type and mutant (S74A, F76A, and R153A) nsp11. The major peaks of wild-type nsp11 and the mutants (S74A, F76A, and R153A) are shown in panel F. Panel F is colored as described for panel D. The sedimentation coefficient (*S*_20,W_) and the calculated molecular weights (MW) are shown in panel G.

In this study, gel filtration chromatography revealed the dimeric architecture of nsp11. Our data indicated that nsp11 eluted primarily in one peak; the calculated molecular mass is approximately 58.9 kDa, which corresponds to a dimer (Fig. 2C, D, and E). This finding is consistent with the dimeric crystal structure of nsp11 (Fig. 1B). The dimerization interface is shown in Fig. 2A and B. Residues Gly1, Leu54, Arg55, Tyr69, Ser74, Phe76, Gly137, Gly138, Arg153, Val165, and Ser166 were chosen as candidate targets to abolish the dimerization. The mutant (G1A, L54A, R55A, Y69A, G137A, G138A, V165A, and S166A) proteins eluted as a dimer; these mutations could not prevent nsp11 dimerization (data not shown). However, elution of the mutant (S74A and F76A) proteins by gel filtration yielded two 280-nm absorption peaks (Fig. 2D and E). Our results indicated that these two mutations significantly disrupt the dimerization in solution. Moreover, the R153A mutant existed mainly as an intermediate form (the calculated molecular mass is approximately 48.8 kDa) compared with the wild type (Fig. 2D and E). Meanwhile, the oligomerization of wild-type and mutant (S74A, F76A, and R153A) nsp11 proteins was further analyzed via sedimentation analytical ultracentrifugation (AUC), and the results were shown in Fig. 2F and G. The molecular weights of monomers and dimers from the wild-type nsp11 protein are approximately 29.2 (approximately 14.04% of the total population) and 63.7 (approximately 86.67%) and are essentially consistent with those of gel filtration chromatography. The sedimentation coefficient (*S*_20,W_) of the mutant (S74A, F76A, and R153A) proteins decreased significantly compared with the wild type, though the relative populations of monomers and dimers of those mutant proteins were not successfully determined. This indicated that the oligomerization of the mutant proteins had markedly changed. Therefore, our biochemical data consistently showed that nsp11 exists mainly as a dimer in solution and that the mutations in the dimerization interface, S74A, F76A, and R153A, disrupt dimerization.

**FIG 3:**
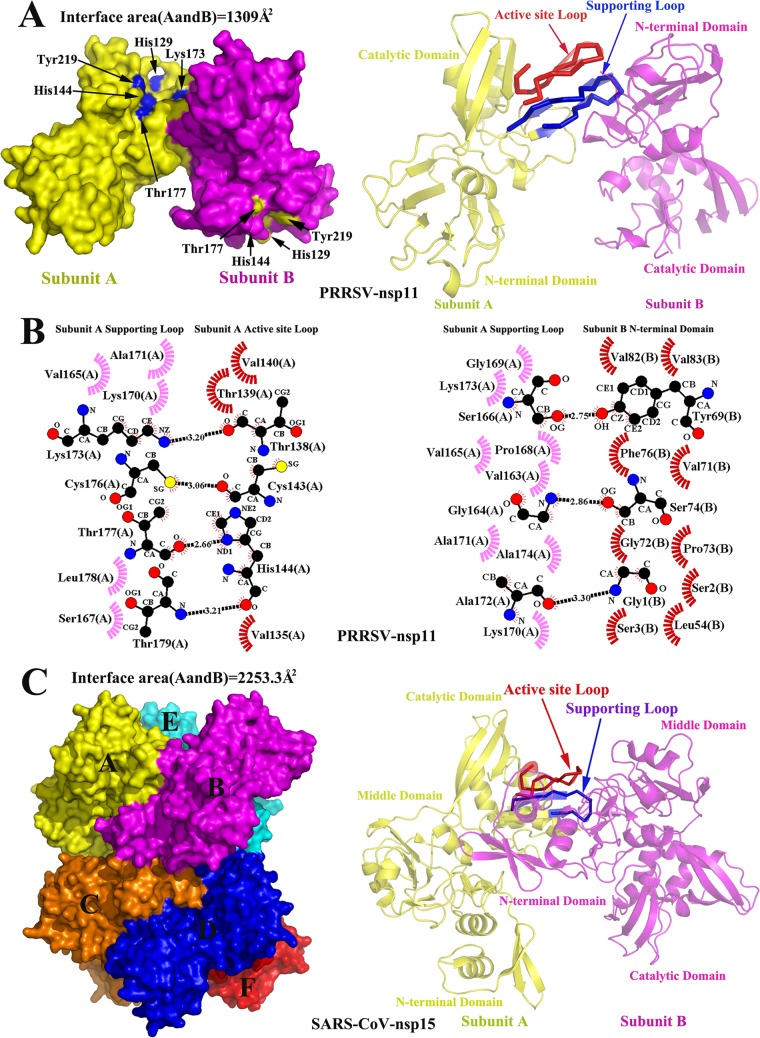
The dimerization mechanism of PRRSV nsp11. (A) Surface representation of dimeric PRRSV nsp11. By analogy with the monomeric structure of SARS-CoV nsp15, the loops consisting of residues His129 to His144 and Val162 to Thr179 are identified as the “active site loop” and “supporting loop,” respectively (23). These two loops are highlighted with red and blue ribbons, respectively (the cartoon transparency was set at 40%). The potential NendoU active sites are shown. (B) Detailed molecular interactions of the supporting loop (subunit A, magenta) with the active site loop (subunit A, red) and the N-terminal domain (subunit B, red) were determined using LIGPLOT. Hydrogen bond interactions and hydrophobic interactions are shown as described for Fig. 2A. (C) Surface representation of hexameric SARS-CoV nsp15 (PDB code 2RHB). The individual subunits are colored and marked A to F. The active site loop (residues His234 to His249) and the supporting loop (residues Lys276 to IIe295) are highlighted as described for panel A. The description of SARS-CoV nsp15 domains is based on a previous report (23). The monomer-monomer buried surface areas of PRRSV nsp11 and SARS-CoV nsp15 were analyzed using PDBePISA.

In our crystal structure, a total binding surface of 1,309 Å^2^ is buried at the interface (Fig. 3A), which is smaller than the subunit A-subunit B binding surface of SARS-CoV nsp15, which is 2,253.3 Å^2^ (Fig. 3C). Regardless, this smaller binding surface may be sufficient to stabilize monomer-monomer interactions because the molecular weight of nsp11 (approximately 25.6) is lower than that of SARS-CoV nsp15 (approximately 38.5). In addition, the catalytic domain of subunit A and the NTD of subunit B are associated with a largely hydrophobic and hydrogen-bonding network (Fig. 2A and B). A total of 16 residues in the NTD of subunit B interact with 17 residues in the catalytic domain of subunit A. Residue Phe76 interacts with residues Tyr150, Leu151, Pro152, Gly164, Val165, and Ser166 via the hydrophobic forces (Fig. 2A and B); thus, Phe76 is a key residue within the dimer interface. Moreover, residue Ser74 interacts with residues Pro152, Val163, and Gly164 and is thus also a key residue within the dimer interface (Fig. 2A and B). In addition, interactional residues Leu77, Val110, and Cys112 were also observed with residue Arg153 (Fig. 2A and B). Therefore, these mutations (S74A, F76A, and R153A) may disturb both hydrophilic and hydrophobic interactions between monomers and prevent the formation of stable dimers.

To clarify the relationship between dimerization and catalytic activity, we performed FRET assays using fluorescence-labeled RNA as the substrate. As predicted, the activity levels of the mutants (S74A and F76A) were significantly decreased (being at least 4-fold less than wild-type levels) but not completely abolished (see Fig. 7C) because the mutant proteins were not purely monomeric. In addition, gel filtration chromatography revealed that the 280-nm absorption peak of the mutant S74A protein was obviously lower than that of the mutant F76A protein with the same amount of total protein (Fig. 2D and E), which indicated that the mutant S74A protein is very unstable. This may be the reason why the NendoU activity of the S74A mutant is lower than that of the F76A mutant. In conclusion, the S74A and F76A mutations severely diminished the catalytic activity, indicating that the dimer is the biologically functional unit.

### The structural basis for nsp11 functioning as a dimer rather than a hexamer.

**FIG 4:**
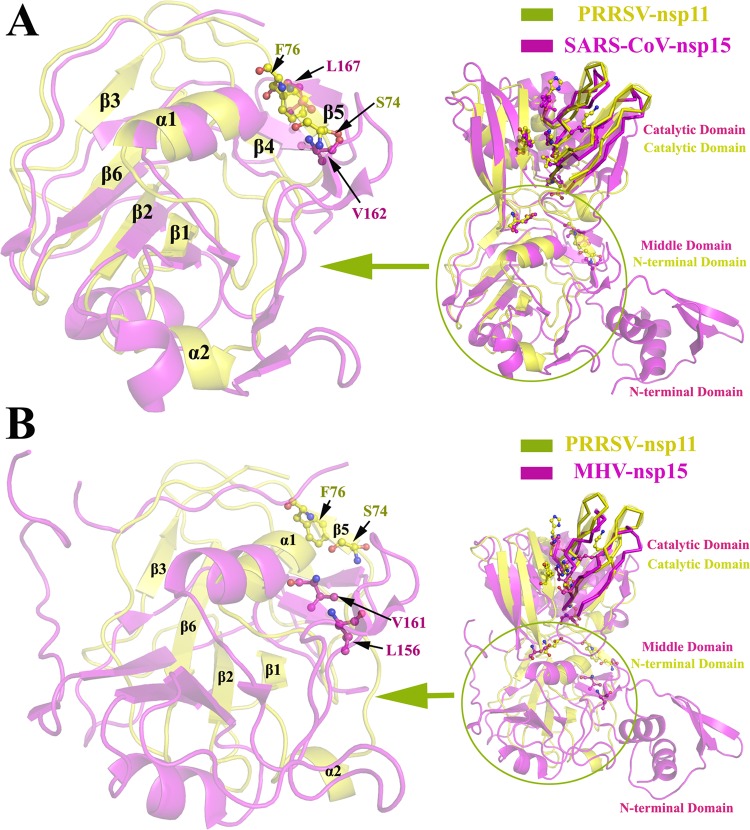
The structural comparison of the N-terminal region of PRRSV nsp11 and the middle region of coronavirus nsp15. (A and B) The structure of PRRSV nsp11 (subunit A, yellow) superimposed onto the structures of SARS-CoV nsp15 (PDB code 2H85, magenta) and MHV nsp15 (PDB code 2GTH, magenta). The structure of the N-terminal region (Gly1 to Gly106) from PRRSV nsp11 superimposed with SARS-CoV nsp15 (Asn62-Ser197) and MHV nsp15 (Ser62-Leu228) is enlarged in panels A and B (the cartoon transparency was set at 60%). The dimerization site determinants Ser74 and Phe76 (corresponding to Val162/Leu156 and Leu167/Val161 in SARS-CoV nsp15 and MHV nsp15, respectively) are labeled with a ball-and-stick (yellow, PRRSV nsp11; magenta, SARS-CoV nsp15 and MHV nsp15) representation. The SARS-CoV nsp15 domains are colored and marked as described for Fig. 3C.

**FIG 5:**
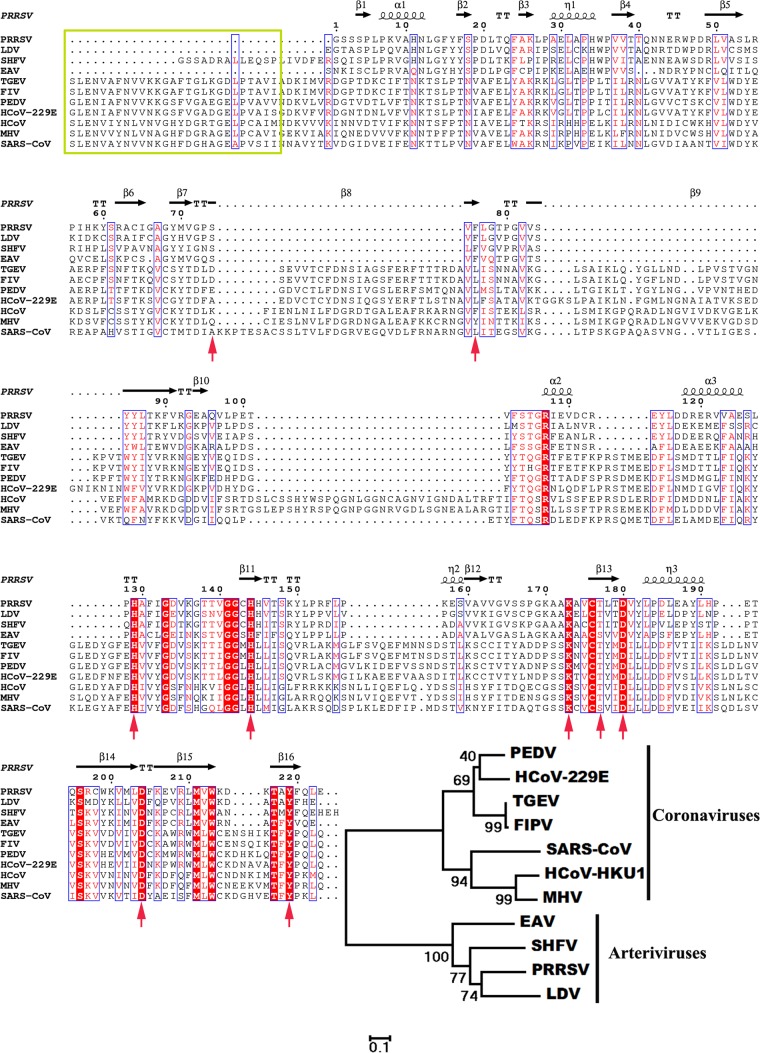
Sequence relationships of arterivirus and coronavirus NendoU domains. The residue numbers at the top refer to the PRRSV nsp11 amino acid sequence. The key residues for potential NendoU activity sites and dimerization sites of PRRSV nsp11 are marked with red arrows at the bottom. The key residues involved in the SARS-CoV nsp15 oligomerization are shown in a yellow frame. Secondary structure elements of PRRSV nsp11 are marked on the top of the alignment (helices with squiggles, β-strands with arrows, and turns with TT letters). The sequences were aligned using ClustalW2, and the alignment was drawn with ESPript 3.0. The phylogenetic relationships were analyzed using the maximum likelihood algorithm in the MEGA package. The different subgenotypes are indicated.

Our crystal structure indicates that nsp11 assembles into dimers, which is different from coronavirus nsp15 (19, 21). The monomer structure of SARS-CoV nsp15 includes three domains, the N-terminal domain (NTD), the middle domain, and the catalytic domain (23) (Fig. 3C); the NTD is critical for hexamerization and interactions with the middle domain and the catalytic domain of an adjacent monomer (19, 23). However, the NTD structure (approximately 61 N-terminal residues) of coronavirus nsp15 is missing in nsp11, and the NTD of nsp11 superimposes onto the middle domain of coronavirus nsp15 (Fig. 4). Moreover, the major determinants of dimerization (Ser74 and Phe76) are significantly different from the key residues involved in the oligomerization of SARS-CoV nsp15 (Fig. 4; Fig. 5), which indicates why the active form of nsp11 is a dimer rather than a hexamer.

In addition, subunits A and B in our crystal structure are highly similar, with a root mean square deviation (RMSD) of 1.18 Å between the 223 Cα atoms, though residues Asp134 to Gly141 and Ser166 to Lys173 are missing in subunit B (Fig. 1C). In analogy with the monomer structure of SARS-CoV nsp15, the loops consisting of residues His129 to His144 and Val162 to Thr179 are identified as the “active site loop” and “supporting loop,” respectively (23). In the dimeric architecture of nsp11, the active site loop and supporting loop from subunit A are packed against one another, and their structures are stabilized by monomer-monomer interactions with subunit B (Fig. 3A). However, the electron density of these loops is missing in subunit B, indicating that they are flexibly disordered. Furthermore, interactions among residues Val135, Thr138 to Val140, Cys143, and His144 in the active site loop and residues Val165, Ser167, Lys170, Ala171, Lys173, and Cys176 to Thr179 in the supporting loop are observed in the structure (Fig. 3B). These extensive interactions between the supporting loop and the adjacent monomer were analyzed. Residues Val163 to Ser166, Pro168, Gly169, and Lys170 to Ala174 from the supporting loop interact with residues Gly1 to Ser3, Leu54, Tyr69, Val71 to Ser74, Phe76, Val82, and Val83 of the adjacent monomer (Fig. 3B), indicating that the supporting loop is involved in dimerization. Therefore, the disappearance of these two loops from subunit B may be attributed to the absence of the adjacent monomer. This finding may explain why nsp11 is fully active as a dimer. Interestingly, the potential link between dimerization and catalytic activity is similar to the mechanism of the functional hexamer of SARS-CoV nsp15 (23). In addition, further research is needed to explore whether the dimer or other oligomers of nsp11 exist in a functional state during arterivirus replication.

### The structure of nsp11 reveals nidovirus-wide conservation of the catalytic domain.

**FIG 6:**
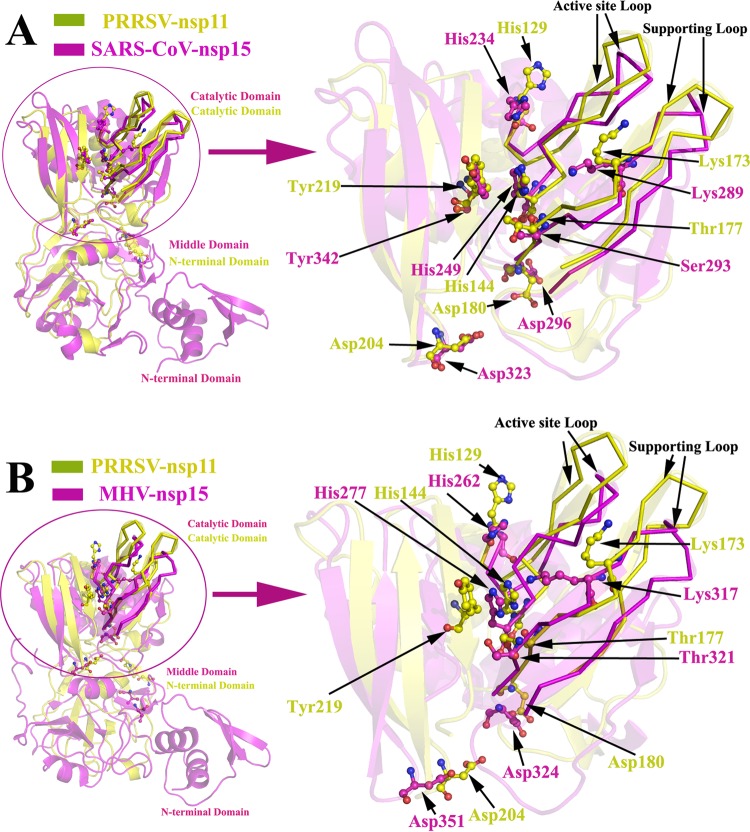
The structure of PRRSV nsp11 reveals the nidovirus-wide conservation of the NendoU domain. (A and B) The structure of PRRSV nsp11 (subunit A, yellow) superimposed onto the structures of SARS-CoV nsp15 (PDB code 2H85, magenta) and MHV nsp15 (PDB code 2GTH, magenta). The structure of the catalytic domain (Ile108-Glu223) of PRRSV nsp11 superimposed with SARS-CoV nsp15 (Asp199-Leu345) and MHV nsp15 (Ser229-Phe369) is enlarged in panels A and B. The potential catalytic active sites are labeled with a ball-and-stick (yellow, PRRSV nsp11; magenta, SARS-CoV nsp15 and MHV nsp15) representation. The “supporting loop” and “active site loop” are highlighted with a ribbon representation (the cartoon transparency was set at 80%) according to structural data for SARS-CoV nsp15 (23). The SARS-CoV nsp15 domains are colored and marked as described for Fig. 3C.

**FIG 7:**
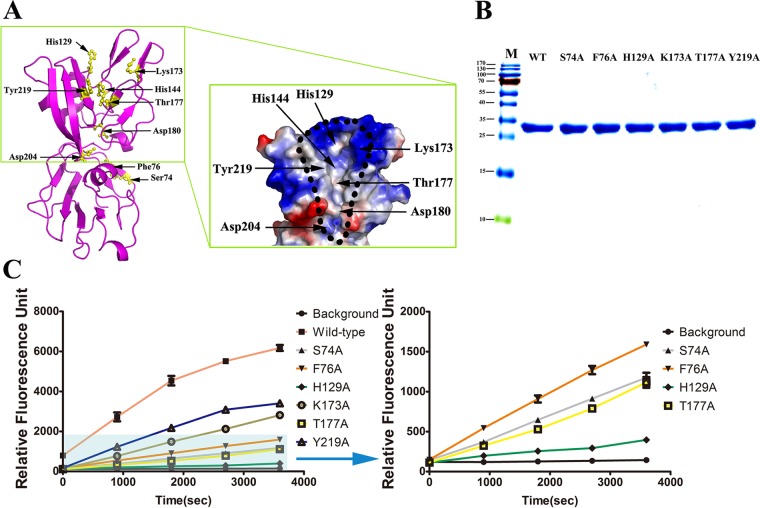
Mutagenesis studies of PRRSV nsp11 endoribonuclease activity. (A) The potential NendoU active sites of nsp11. The potential NendoU active sites and dimerization site determinants (Ser74 and Phe76) are labeled with a ball-and-stick (yellow) representation. The molecular surface model is colored as described for Fig. 1D and E. The putative nuclease active center is highlighted with a black dashed line. (B) SDS-PAGE analysis of wild-type and mutant (S74A, F76A, H129A, K173A, T177A, and Y219A) nsp11. Molecular weight markers are shown. (C) FRET-based enzyme activity assay. Wild-type and mutant (S74A, F76A, H129A, K173A, T177A, and Y219A) nsp11 endoribonucleases are labeled with different colors. The values (± SD) of the results of triplicate experiments are shown.

Multiple-sequence alignment indicated that the amino acid sequence identity between arterivirus nsp11 and coronavirus nsp15 is only approximately 16.1% to 25.1%, as demonstrated by their distance on the evolutionary tree (Fig. 5). Moreover, there are distinct differences between the NTD of nsp11 and the middle domain of coronavirus nsp15 (the RSMDs with SARS and MHV are 2.41 and 2.79, respectively) (Fig. 4). However, the structures of the catalytic domains can be nearly perfectly superimposed (the RSMDs with SARS and MHV are 2.14 and 2.09, respectively), especially in the active site loop and supporting loop regions (Fig. 6). Additionally, the structural comparison demonstrated that residues His129, His144, Lys173, Thr177, Asp180, Asp204, and Tyr219 from nsp11 superimpose well onto the corresponding residues of coronavirus nsp15 (Fig. 5 and 6), indicating the relative conservation of key active site residues and similar endoribonuclease cleavage mechanisms shared among nidoviruses (families Arteriviridae and Coronaviridae). In this study, endoribonuclease activity of the wild-type and mutant nsp11 protein was measured, and the results are shown in Fig. 7C. Enzyme activity assays for the wild-type and H129A, K173A, T177A, and Y219A mutant proteins were performed under identical conditions, and the activity levels of the mutants were significantly reduced compared with the wild-type level (Fig. 7C), indicating that these residues are located in important NendoU active sites.

Previous results suggested that the catalytic mechanism of the nidovirus endoribonuclease could be consistent with an RNase A-like reaction mechanism that cleaves the RNA substrate to form a 2′,3′-cyclic phosphodiester and a 3′-phosphomonoester by transphosphorylation and hydrolysis (20). The catalytic His129 and His144 residues of nsp11 (corresponding to His234 and His249 of SARS-CoV nsp15) are thought to accept and donate protons during production of the 2′-3′-cyclic phosphate (19), which may explain why the catalytic activity of the H129A mutant is much lower than that of the K173A mutant. Previous studies also demonstrated that residues His129, His144, and Lys173 (corresponding to His234/His262, His249/His277, and Lys289/Lys317 in SARS-CoV nsp15 and MHV nsp15, respectively) are essential for endoribonuclease activity in coronaviruses and arteriviruses (19–22, 35). In our crystal structure, these three putative catalytic residues (His129, His144, and Lys173) surround a positively charged cavity in the catalytic domain, with Thr177 located in the middle of the groove (Fig. 7A). Thr177 (corresponding to Ser293 and Thr321 in SARS-CoV nsp15 and MHV nsp15, respectively) could also be important for substrate recognition and binding (19, 36, 37). Moreover, Tyr219 (corresponding to Tyr342 in SARS-CoV nsp15) has been implicated in the orientation and binding of the substrate (19, 36). As predicted, the catalytic activity levels of the T177A and Y219A mutants were significantly decreased but not completely abolished.

**FIG 8:**
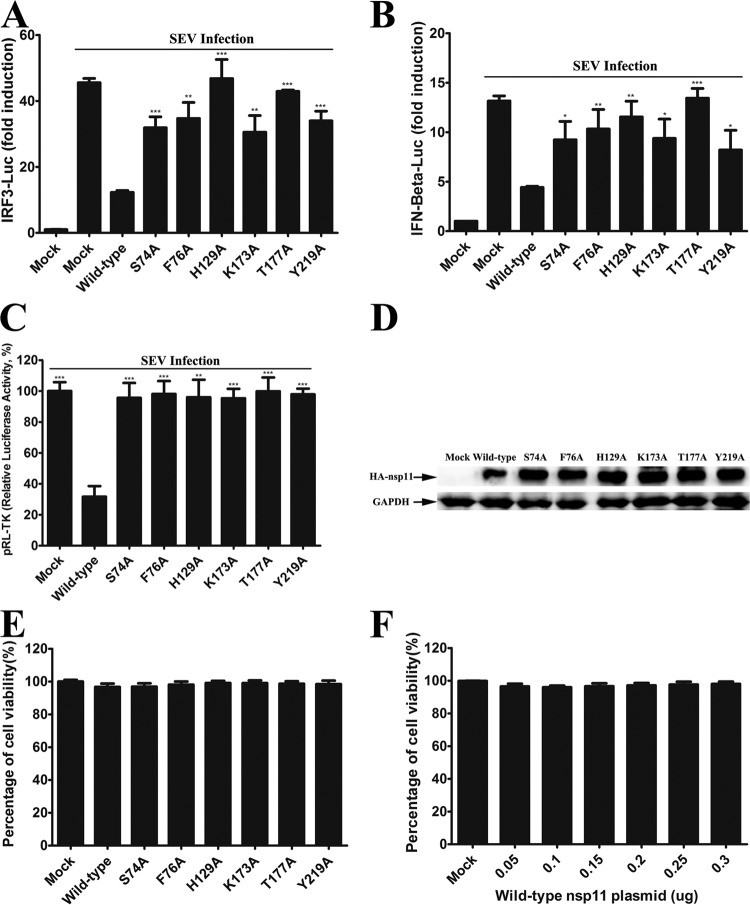
The potential cytotoxicity of wild-type nsp11 may inhibit IFN-β promoter activation. (A and B) HEK293T cells were cotransfected with IRF3-Luc plasmid (0.1 μg) (A) of IFN-β–Luc (B) and pRL-TK plasmid (0.02 μg), together with the wild-type and mutant nsp11 plasmids (0.4 μg). At 24 h after the initial transfection, the cells were infected with Sendai virus (SEV). At 40 h posttransfection, the cells were harvested and the luciferase activity was measured. The firefly luciferase activity was normalized to Renilla reniformis luciferase, and the untreated empty vector control value was set to 1. *, *P* < 0.05 (considered significant compared with the luciferase activity of the cells expressing the wild-type protein); **, *P* < 0.01 (considered highly significant); ***, *P* < 0.001 (considered extremely significant). (C) Relative luciferase activity of pRL-TK. Relative luciferase activity = 100 × (the luciferase value for the wild-type and mutant strains/the luciferase value for the control group). (D) Western blot analysis of the expression levels from wild-type and mutant nsp11. (E and F) Cell viability analysis of wild-type and mutant nsp11 in HEK293T cells. Cell viability of wild-type and mutant nsp11 was evaluated via the use of a CellTiter-Glo luminescent cell viability assay. Percent cell viability = 100 × (luminescence of the experimental group/luminescence of the control group).

In addition, previous studies reported that the endoribonuclease activity of PRRSV nsp11 is essential to inhibit IFN-β induction (38). We found that the overexpression of wild-type nsp11 markedly inhibited the activity of the IFN-β luciferase reporter induced by Sendai virus (SEV), while the mutants (S74A, S76A, H129A, K173A, T177A, and Y219A) lost the capacity to block the activation of IFN-β promoter (Fig. 8A and B). Because the recombinant arterivirus nsp11 protein displays broad substrate specificity *in vitro* and is extremely toxic to prokaryotic and eukaryotic cells (20), it is possible that the suppression of IFN-β induction by wild-type nsp11 is due to its cytotoxicity. HEK293T cells expressing wild-type nsp11 appeared to be in good shape and showed no obvious cytotoxicity in detection experiments performed with the CellTiter-Glo luminescent cell viability assay (Fig. 8E and F). However, when we analyzed the ability of wild-type and mutant nsp11 to inhibit IFN-β induction by the use of a Dual-Luciferase reporter assay system (Promega), we found that the value for pRL-TK, an internal control reporter, was significantly lower in cells expressing wild-type nsp11 than in cells expressing nsp11 mutants or in cells that had received mock treatment (Fig. 8C), indicating that wild-type nsp11 inhibits host gene expression. Coincidentally, none of the tested nsp11 mutants without cytotoxicity significantly inhibited IFN-β induction (Fig. 8A, B, and C). Therefore, we could not exclude the possibility that the potential cytotoxicity of wild-type nsp11 inhibits IFN-β induction. It should be noted that this study involved the individual expression of nsp11, outside the context of infection. Whether the endoribonuclease function of nsp11 specifically contributes to the decline of innate immune functions in PRRSV infection requires further investigation.

### Conclusions.

In summary, we provide the first structural information for arterivirus endoribonuclease nsp11, which has a novel dimeric structure that dramatically distinguishes it from coronavirus nsp15. Our biochemical data showed that mutation of key residues (Ser74 and Phe76) in the dimerization interface disrupts dimerization in solution and markedly impairs the endoribonuclease activity *in vitro*. Furthermore, structural analyses showed that the absence of adjacent monomer interactions might damage the structural stability of the catalytic domain, which indicates why the biologically active unit of nsp11 is a dimer. Furthermore, structural conservation of the catalytic domain in members of the order Nidovirales (families Arteriviridae and Coronaviridae) was also observed in this study. These results provide a model that will contribute to an understanding of the structure-function relationship of endoribonucleases in the order Nidovirales, and our findings will serve as a structural basis for the development of new nsp11-specific antiviral drugs and other inhibitors.
